# A Co-Operative Tour to Rome

**Published:** 1893-05-20

**Authors:** 


### A Co-operative Tour to Rome.

We have received from Mr. Holdsworth Lunn details of a series of parties which he proposes to organise in September next for the purpose of enabling medical men, accompanied, if desired, by their families, to visit Rome for the Medical Congress. Mr. Lunn has assisted his brother, the Rev. Dr. Lunn, and Mr. Woolrych Perowne in the recent highly successful tour which these gentlemen organised for the clergy of different denominations. Four hundred and fifty ladies and gentlemen, travelling on different days, in parties numbering from sixty to a hundred, availed themselves of Dr. Lunn's arrangements, which included a series of lectures by the Rev. Professor Mahaffy and the Rev. H. R. Haweis on "Ancient and Modern Rome."

The autumn tour is planned on the same lines, and is calculated at a cost of twenty guineas for each person. The party leaves London on September 18th, and proceeds to Rome, where they arrive September 22nd, viâ Basle, Lucerne, and Milan, passing through the Saint Gothard Tunnel. Ten days will be spent at Rome, covering the time of the Medical Congress, and the return is made viâ Florence and Basle to London, which is to be reached on October 5th. The twenty guineas will cover second-class railway fare and first-class hotel accommodation for the whole period. Special supplementary tours to Naples and Pompeii, to Venice, and to the Italian lakes have been arranged at an absurdly small additional cost, and members of the party will be permitted to break the journey at any of the principal stations on the return journey, returning at any time within forty-five days. The whole scheme illustrates in an extraordinary manner the advantages of co-operation. Special pains have been taken to minimise its disadvantages, since, though every aid in making plans will be afforded to members of the party, no attempt will be made to take them about in bands on their arrival in Rome or to hamper individual tastes. The only exception to this rule is a proposed expedition to the Catacombs, under the guidance of Dr. Russell Forbes, and those who have made the experiment of visiting the Catacombs alone will be able to appreciate to the full the advantages on this occasion of a little cheerful company. Medical men desirous of combining a visit to the Congress with a holiday free from worry and extortionate expense will no doubt be glad to avail themselves of the proposed scheme, of which full particulars may be learned by application to the Secretary, "Review of the Churches," 5, Endsleigh Gardens, London, N.W.

